# Characterization of Centromeric Histone H3 (CENH3) Variants in Cultivated and Wild Carrots (*Daucus* sp.)

**DOI:** 10.1371/journal.pone.0098504

**Published:** 2014-06-02

**Authors:** Frank Dunemann, Otto Schrader, Holger Budahn, Andreas Houben

**Affiliations:** 1 Julius Kühn-Institut (JKI) - Federal Research Centre for Cultivated Plants, Institute for Breeding Research on Horticultural Crops, Quedlinburg, Germany; 2 Leibniz-Institute of Plant Genetics and Crop Plant Research (IPK), Chromosome Structure and Function Laboratory, Gatersleben, Germany; National Cancer Institute, United States of America

## Abstract

In eukaryotes, centromeres are the assembly sites for the kinetochore, a multi-protein complex to which spindle microtubules are attached at mitosis and meiosis, thereby ensuring segregation of chromosomes during cell division. They are specified by incorporation of CENH3, a centromere specific histone H3 variant which replaces canonical histone H3 in the nucleosomes of functional centromeres. To lay a first foundation of a putative alternative haploidization strategy based on centromere-mediated genome elimination in cultivated carrots, in the presented research we aimed at the identification and cloning of functional CENH3 genes in *Daucus carota* and three distantly related wild species of genus *Daucus* varying in basic chromosome numbers. Based on mining the carrot transcriptome followed by a subsequent PCR-based cloning, homologous coding sequences for CENH3s of the four *Daucus* species were identified. The ORFs of the CENH3 variants were very similar, and an amino acid sequence length of 146 aa was found in three out of the four species. Comparison of *Daucus* CENH3 amino acid sequences with those of other plant CENH3s as well as their phylogenetic arrangement among other dicot CENH3s suggest that the identified genes are authentic CENH3 homologs. To verify the location of the CENH3 protein in the kinetochore regions of the *Daucus* chromosomes, a polyclonal antibody based on a peptide corresponding to the N-terminus of *DcCENH3* was developed and used for anti-CENH3 immunostaining of mitotic root cells. The chromosomal location of CENH3 proteins in the centromere regions of the chromosomes could be confirmed. For genetic localization of the CENH3 gene in the carrot genome, a previously constructed linkage map for carrot was used for mapping a CENH3-specific simple sequence repeat (SSR) marker, and the CENH3 locus was mapped on the carrot chromosome 9.

## Introduction

The cultivated carrot (*Daucus carota*) is one of the most important vegetable plants in the world. With a current annual world production of more than 30 million tons and a total growing area of about 1.5 million hectares (FAOSTAT 2012) it ranks among the top ten vegetable crops. Carrot is the most widely grown species of the genus *Daucus,* a member of the large and complex Apiaceae plant family. The genus *Daucus* includes around 25 species and was subdivided taxonomically into five [1], and later into seven sections [2], but both classification systems are not yet fully congruent with molecular phylogenetic studies [3]. *Daucus* species are widespread in the temperate areas of the northern hemisphere, but few species exist also in South America and Australia [3]. *D. carota* is a diploid outcrossing species with nine chromosome pairs (2n = 2x = 18). *D. capillifolius*, *D. sahariensis* and *D. syrticus* are the other members of the genus with 2n = 18 chromosomes, whereas *D. muricatus* (2n = 20) and *D. pusillus* (2n = 22) have a slightly higher chromosome number. It is assumed that x = 11 is the basic chromosome number in Apiaceae family, and x = 10 and x = 9 are its derivatives [4]. However, a few polyploid species as for example *D. glochidiatus* (2n = 4x = 44) and *D. montanus* (2n = 6x = 66) also exist.

The haploid genome size of carrot has been estimated at 473 Mbp [5], which is similar to rice. First carrot linkage maps have been developed based on several types of molecular markers [6], [7], and a BAC library of the carrot genome has been created [8]. Furthermore, the carrot transcriptome has been revealed recently by next generation sequencing (NGS) technology [9]. Carrot is also well known as a model species for gene transfer using both genetic modifications by vector and non-vector methods, which is a major prerequisite for functional gene studies [10].

Despite all these progressed molecular and biotechnological developments comparatively limited work has been done on the cytological and molecular-cytogenetic characterization of the carrot genome. Individual carrot chromosomes are small and uniform in shape and length [11] and are therefore a difficult object for cytogenetic research. Using rDNA genes as probes for fluorescence *in situ* hybridization (FISH) analysis, chromosomal karyotypes were developed for cultivated carrots and other Apiaceae species [11], [12]. Carrot BAC clones were used to integrate genetic and physical maps based on pachytene chromosomes of *D. carota*, and mitotic chromosomes of two further 22-chromosome *Daucus* species as well [13].

As a cross-pollinated species suffering from inbreeding depression carrot provides some challenges in plant (hybrid) breeding. Due to the biannual nature of carrots and the difficulties to produce sufficient amounts of seed from selfings, the generation of genetically homogeneous genotypes with a high degree of homozygosity is a long lasting and inefficient task in carrot breeding programs. As an alternative and/or supplement to traditional inbred line production in carrots, double-haploid plants might be produced by *in vitro-*regeneration of plants through anther or microspore culture. However, haploid production by tissue culture techniques is generally highly genotype-dependent and has been reported to be very inefficient in Apiaceae species [14]. The generation of doubled haploids using naturally occurring mechanisms of uniparental genome elimination induced by interspecific hybridization has not yet been reported for *Daucus*.

Recently, a breakthrough technology has been presented by Ravi and Chan [15], [16], which uses centromere-mediated genome elimination processes for the generation of haploid and double-haploid plants. It was demonstrated for the first time in *Arabidopsis thaliana*, that haploids can be generated through manual cross-fertilizations after manipulating a single centromere protein, the centromere-specific histone H3 variant CENH3, in one of the parents designated as ‘haploid inducer’ [15]. Uniparental genome elimination using this strategy was suggested to function in any (crop) plant due to the universal centromere mechanism based on CENH3 function [15], [16].

In eukaryotes, centromeres are the assembly sites for the kinetochore, a multi-protein complex to which spindle microtubules are attached at mitosis and meiosis, thereby ensuring segregation of chromosomes during cell division [17]. They are specified by incorporation of CENH3, which replaces canonical histone H3 in the nucleosomes of functional centromeres [18]. Modifications in CENH3 gene transcription or translation could affect the ability to assemble intact CENH3 chromatin and might result in the loss of CENH3 from the centromere region and a loss of proper centromere function. Contrary to canonical histone H3, which is extremely conserved in eukaryotes, CENH3 shows considerable variability between species and shows some signs of adaptive evolution [19]. Presently, investigations on structure and function of plant CENH3s have been reported for a variety of species originating from at least 20 different plant genera. Among them there are most important cereals such as *Zea mays* [20], *Oryza sativa* [21], [22], *Saccharum officinarum* [23], *Hordeum* species [24] and a few other monocots including vegetable *Allium* species [25]. Besides, CENH3s have been intensively studied in the model dicot species *Nicotiana tabacum* [26], some *Brassica* species [27] and several members of the Leguminosae family including soybean, common bean, and peas [28]–[31]. To our knowledge, no investigation on CENH3s from Apiaceae species has been reported up to now.

To lay a first foundation of a putative alternative haploidization strategy based on centromere-mediated genome elimination in cultivated carrots, the major aim of the present study was to identify functional *Daucus* CENH3 genes and to verify the location of the CENH3 protein in the kinetochore regions of the *Daucus* chromosomes. Complementary coding sequences of CENH3s of four *Daucus* species were identified and phylogenetically compared with previously reported plant CENH3s. A generated polyclonal CENH3 antibody confirmed the centromeric location of CENH3 proteins, and the CENH3 locus was genetically mapped on the carrot chromosome 9.

## Materials and Methods

### Plant Material and Isolation of Genomic DNA and cDNA

The carrot (*D. carota* subsp. *sativus*) cultivar ‘Deep Purple’ (DP) and one accession each from the Mediterranean wild species *D. muricatus* (2n = 2x = 20, accession W243/06), the South American species *D. pusillus* (2n = 2x = 22, accession 989/92–3) and the Australian species *D. glochidiatus* (2n = 4x = 44, accession DAL 341/00) were used for this study. Seeds of wild species were originally received from Hortus Botanicus Coimbra, Portugal (*D. muricatus*), Plant Science Laboratory, University of Reading, U.K. (*D. pusillu*s) and Warwick Genetic Resources Unit, Warwick University, Wellesbourne, U.K. (*D. glochidiatus*), and have been kindly provided by T. Nothnagel (Julius Kühn-Institut, Quedlinburg, Germany). Plants obtained from seeds were grown in pots in a greenhouse for DNA and RNA isolations. Total genomic DNA from young leaf tissue of individual plants was extracted using the Qiagen DNeasy Plant Mini kit (Qiagen, Hilden, Germany) following the manufacturer’s instructions. For RNA isolation, small leaflets were immediately frozen in liquid nitrogen and ground to fine powder by using a swing mill. Total RNA was isolated by using the Qiagen RNeasy Plant Mini kit. An additional DNAse step (Qiagen) was included in this procedure. The qualitatively and quantitatively checked RNA solution was then used to synthesize cDNA with the RevertAid First Strand cDNA Synthesis Kit (Thermo Fisher Scientific, St. Leon-Rot, Germany).

### Identification and Cloning of CENH3 Genes

To identify *D. carota* CENH3 orthologous sequences, the assembled carrot transcriptome [9] was used for *in silico* gene mining. A Fasta file containing 58,751 sequences was loaded into the software BioEdit version 7.0.5.3. [32] and screened by NCBI Local BLAST [33] using the tBlastn search option and the translated amino acid sequence of *Nicotiana tabacum* CENH3 (GenBank number BAH03515, [26]) as a query. Since the beginning of the gene was not found, a degenerate PCR forward primer (DCEN1-F: 5′- atg gcg aga acn aar cay) based on the first six amino acids at the N-terminal region of CENH3s of four different dicot species (*A. thaliana*, *Brassica rapa*, *N. tabacum* and *Glycine max*) and an internal gene-specific reverse primer designed from the contig representing the last part of the putative carrot CDS (DCEN1-R: 5′- acg gag cag cag gaa tta ga) were designed and used for PCR-based cloning of the missing carrot CENH3 CDS region. DNA fragments with the expected size of approximately 240 bp obtained after PCR with cDNA templates of ‘DP’ were excised from the gel, purified using the MinElute Gel Extraction Kit (Qiagen, Hilden, Germany) and cloned by the pGEM-T Easy Vector System (Promega, Madison, USA). Plasmid inserts of selected clones were sequenced by Eurofins-MWG-Operon (Ebersberg, Germany). Based on the sequences obtained a second round of screening the *Daucus* transcriptome was performed with the tBlastx program, and two additional short contigs representing the beginning of the CENH3 gene were detected including a part of the 5′-UTR region in one of the contigs, which was used to design a new PCR primer (DCEN2-F: 5′- ccg tta gaa atc acg gtc atc a). Using this primer together with a newly created reverse primer exactly fitting the last nucleotides of the CDS (DCEN2-R: 5′ - acc agg gct gcg ctt tct) we were able to amplify the complete *Daucus* CENH3 coding region as well as its full-length genomic sequence. For latter approach, Long Range PCR (LR-PCR) based on the ‘Long PCR Enzyme Mix’ (Thermo Scientific) was carried out with genomic DNA of *D. carota* cv. ‘DP’. PCR fragments with a size of about 4.5 kb were cloned using the pGEM-T Easy Vector System (Promega). Clones were sequenced by Eurofins-MWG-Operon using the sequencing primers M13uni(-21) and M13rev(-49) for sequencing the beginning and the end of the cloned inserts. To obtain the full-length nucleotide sequence a primer walking approach based on five intermediate primers was used. At least two replications of plasmid insert sequencing were performed for each clone to provide a sufficient reading confidence required for accurate manual assembling of a consensus gene sequence.

For sequence alignment and phylogenetic analysis, putative amino acid sequences were deduced from the determined *Daucus* cDNA sequences and compared with a selection of published CENH3 proteins from other plant species and canonical histone H3 of *A. thaliana* as an outgroup. Multiple sequence alignment (MSA) of CENH3 proteins was performed by ClustalW using the Lasergene software package (DNASTAR, Madison, WI, USA). A phylogenetic tree was constructed using the Kimura distance formula to calculate distance values and bootstrap analysis (10,000 replicates).

### Transcriptional Analysis of CENH3 Gene Expression

For the development of species-specific PCR primers, final CENH3 CDS nucleotide sequences were aligned by ClustalW (Lasergene), and based on sequence differences detected among *D. carota* and *D. glochidiatus*, a set of two PCR primer pairs (named as DcEXP and DpgEXP) was developed for testing transcription activity of parental CENH3s by reverse transcription (RT) - PCR (for primer sequences, see Figure S3A). As reference gene for RT-PCR the constitutive (house-keeping) gene *β-actin* was chosen, and the following primers were used: DcACT-F: 5′- aca ctg gtg tga tgg ttg ga; DcACT-R: 5′-tgg tga taa ctt gcc cat ca [34]. RT-PCR was carried out in a total volume of 25 µl containing 1 µl of the synthesized cDNA solution, 1 U of ‘DreamTaq’ DNA polymerase (Thermo Fisher Scientific), 1x *Taq* polymerase buffer with MgCl_2_ (Thermo Fisher Scientific), 0.2 µM of each primer and 0.2 mM of each dNTP. Amplification conditions were as follows: 1 cycle of 3 min at 94°C; 35 cycles of 94°C for 30 sec, 53°C (DcEXP, DpgEXP) or 57°C (DcACT) for 45 sec, 72°C for 1 min; final extension of 72°C for 5 min. A positive (genomic DNA) and a negative control (water) were included into RT-PCR.

### Linkage Mapping

Based on a SSR (simple sequence repeat) sequence found within an intron of the cloned genomic *D. carota* CENH3 sequence, a PCR primer pair (DCEN-SSR-F: 5′- ggt ctc tct ccc tca cac act t; DCEN-SSR-R: 5′- cgt ctc gga gtt ccc tgt ata a) was designed and used for linkage mapping. For chromosomal location of the carrot CENH3 gene a genetic map constructed previously for the carrot progeny DM19 was used [Budahn, unpublished]. DM19 was developed from an initial cross of two parental *D. carota* leaf mutants (‘Yellow’ and ‘Cola’). Selected F_1_ plants were self-pollinated to produce the F_2_ generation used for linkage mapping. The genetic map has been constructed on a basis of 161 individual DM19 plants and includes 285 molecular markers located on nine linkage groups [Budahn, unpublished]. SSR analysis was carried out according to the PCR conditions published for carrot SSRs [6] using a LI-COR 4300 DNA analyzer (LI-COR Biosciences, Lincoln, NE, USA). DNA fragments polymorphic for the parents were scored in the DM19 progeny, and marker scores were converted to the segregation type codes required for linkage mapping with the JoinMap version 4.0 software [35]. Linked loci were grouped using LOD thresholds from 5.0 to 10.0 in steps of 0.2 and recombination frequency ≤0.4. The jump threshold was set to 5.0 and the third mapping round was carried out. Map distances in centi-Morgan (cM) were calculated using the Kosambi function.

### Immunostaining

Based on a peptide corresponding to the N-terminus of *DcCENH3* (NH_2_-RTKHPAKRTSGHRSRGPPLS-CONH_2_; amino acids 3–22) polyclonal IgG antibodies were generated. Peptide synthesis, immunization of three rabbits and affinity purification of *Daucus* CENH3-antiserum on sepharose columns was performed by Pineda Antikörper-Service (Berlin, Germany). In addition, a commercially available mouse antibody to α-tubulin (clone DM 1A, Sigma) was used. A Cy3-conjugated anti-rabbit IgG (Dianova) and an anti-mouse Alexa 488 antibody (Molecular Probes) were used as secondary antibodies. Immunostaining was performed on slides prepared from root tips of *D. carota* and *D. glochidiatus.* Seeds were germinated on moist filter paper at room temperature in dark for 3 days. Prior to incubation with either antibody root tips (1.5–2 cm) were fixed 5 min under mild vacuum at room temperature and 25 min on ice in freshly prepared 3.7% paraformaldehyde solution (PFA) containing phosphate-buffered saline (1xPBS, pH 7.3) and then washed three times for 5 min in 1x PBS on ice. For immunostaining with anti- α-tubulin antibody, material was fixed in 3.7% PFA solution containing microtubules stabilizing buffer (1xMTSB prepared with 50 mM Pipes, 2 mM EGTA, 2 mM MgSO_4_). Meristematic regions of root tips were digested by treating with an enzyme mix (2 vol enzyme mixture: 0.7% cellulase (Calbiochem), 0.7% cellulase R10 (Duchefa), 1% pectolyase (Sigma), 1% cytohelicase (Sigma) plus 1 vol 1x PBS/MTSB, pH 7.5) at 37°C until the material became soft (about 30–40 minutes). The macerated material was shortly washed and then squashed in PBS or MTSB on a slide. Coverslips were removed using liquid nitrogen and slides were immersed in 1x PBS/MTSB and further processed on the same day or the day after. The slides were incubated for 1 h at 37°C in a moisture chamber with blocking solution (3% BSA in 1x PBS/8% BSA in 1xMTSB, 0.1% Tween 20), followed by an incubation at 10°C overnight with the primary antibody diluted in 1xPBS/MTSB supplemented with 1% BSA. Dilutions were 1∶500 for anti-CENH3, and 1∶100 for antibody to α-tubulin. Following three washes in 1xPBS/MTSB for 5 min the secondary antibody (anti-rabbit-Cy3 diluted 1∶300 in 1xPBS/MTSB supplemented with 1% BSA or anti-mouse-Alexa 488 diluted 1∶200 was applied for 45 min at 37°C. After 3 final washes with PBS buffer 5 min each time, the slides were counterstained with 4′,6-diamino-2-phenylindole (DAPI) and mounted in Vectashield mounting medium (Vector Laboratories, Burlingame, CA, USA). In double immunostaining experiments (CENH3 and α-tubulin) the two primary or secondary antibodies were incubated together.

## Results and Discussion

### Identification of *Daucus* CENH3 and Phylogenetic Analysis

The amino acid sequence of *N. tabacum* CENH3 [26] was used as a query in a tBlastn search against the assembled carrot transcriptome [9]. After bioinformatic CENH3 mining, two overlapping contigs were identified which represented about 85% of the whole putative CENH3 coding sequence (CDS). Since the highly variable N-terminal region was not found, an intermediate PCR-based cloning step was performed. Finally, four overlapping contigs were found in the *D. carota* transcriptome representing the whole CENH3 coding sequence including a part of the 5′-UTR region. Based on the assembled sequence, specific primers were designed for PCR-based cloning of the complete *Daucus* CENH3 coding region. The *D. carota* homolog of CENH3 was named *DcCENH3* (GenBank number KJ201903) and has been identified with a nucleotide sequence length of 438 bp encoding a 146 amino acid (aa) protein, which is one of the shortest plant CENH3s known so far. Similarly, the ORFs of *D. pusillus* (*DpCENH3*, KJ201905), *D. glochidiatus* (*DgCENH3*, KJ201906) and *D. muricatus* (*DmCENH3*, KJ201904) were isolated. *DpCENH3* and *DgCENH3* also showed a DNA size of 438 bp, whereas *DmCENH3* cDNA was 3 bp shorter (435 bp, 145 aa). To our knowledge, the cloned genes from different carrot species are the first CENH3s isolated from the large Apiaceae plant family.

**Figure 1:**
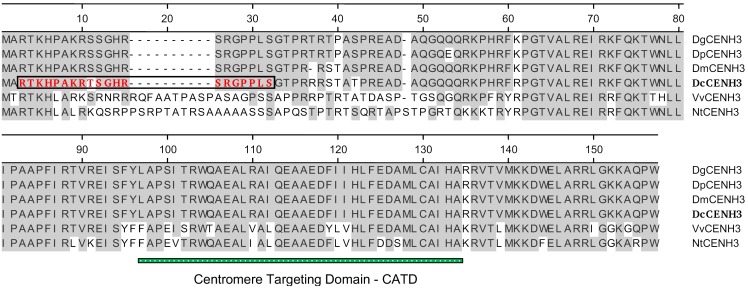
Multiple sequence alignment of the deduced *Daucus* CENH3 proteins and comparison with CENH3 sequences from *Nicotiana tabacum* (GenBank accession number BAH03515) and *Vitis vinifera* (XP_002281073) showing the highest similarity to *Daucus* CENH3s after multiple alignment of various plant CENH3 proteins (see **Figure 2**). Sequences were compared by ClustalW (Lasergene). The putative centromere targeting domain (CATD) spanning loop 1 and α-2 helix is marked by a crossbar. The position used for construction of a peptide antibody against *DcCENH3* is boxed.

A multiple sequence alignment of the nucleotide sequences is shown in Figure S1, and the amino acid sequences deduced from the ORFs are shown in Figure 1, respectively. Except for the unique feature of the missing triplet in *D. muricatus* the CENH3 sequences of the four species differed only by a few nucleotides. The highest similarity was found between *DpCENH3* and *DgCENH3* with 98.4% nucleotide identity (Figure S2) resulting in an exchange of a single amino acid (Figure 1). In each comparison among the *Daucus* species the homology was higher than 95% identity on a nucleotide level, with a maximum of six amino acid changes between *D. carota* and *D. glochidiatus*. With regard to the putative centromere targeted domain (CATD), the protein sequences were identical (Figure 1). The nearly identical CENH3 variants in the Australian accession of *D. glochidiatus* (2n = 4x = 44) and the American representative of *D. pusillus* (2n = 2x = 22), which both have the basic chromosome number of x = 11, indicate the putatively close phylogenetic relationship between these two species. Molecular taxonomic studies have placed both species in the same *Daucus II* subclade [36], but it is unknown if polyploid *D. glochidiatus* is the result of a recent hybridization with any of the diploid species investigated in this study. Because of the fragmentary knowledge on systematics and phylogeny of the genus there is no indication yet either for a potential *Daucus* ancestor or a hypothetical ancestral karyotype [12].

To analyse the intron/exon structure of *Daucus* CENH3, the full-length genomic sequence of *D. carota* CENH3 was amplified with the same primer pair (DCEN2) used for the cDNAs, cloned into plasmids and sequenced by a primer walking approach. A single sequence was obtained, with a total length of 4,515 bp. Alignment of the *D. carota* CENH3 cDNA sequence with the genomic sequence resulted in a gene structure consisting of 7 exons and 6 introns of very different sizes (Figures S3A and S3B). A similar structure of 7 exons and 6 introns was observed for rice CENH3 genes [22], and 7 exons were also reported for *Brassica nigra* [27]. Exon 2 of carrot CENH3 was found to be extremely short (14 bp), whereas intron 5 displayed a sequence length of 2,354 bp, which is more than 50% of the whole gene. Exon 3 of *DcCENH3* contains the 3 nucleotides C-G-A (coding for arginine), which are missing in the *DmCENH3* CDS, but their position inside exon 3 indicates, that no alternative splicing has caused the loss of this single triplet in *D. muricatus.*

**Figure 2:**
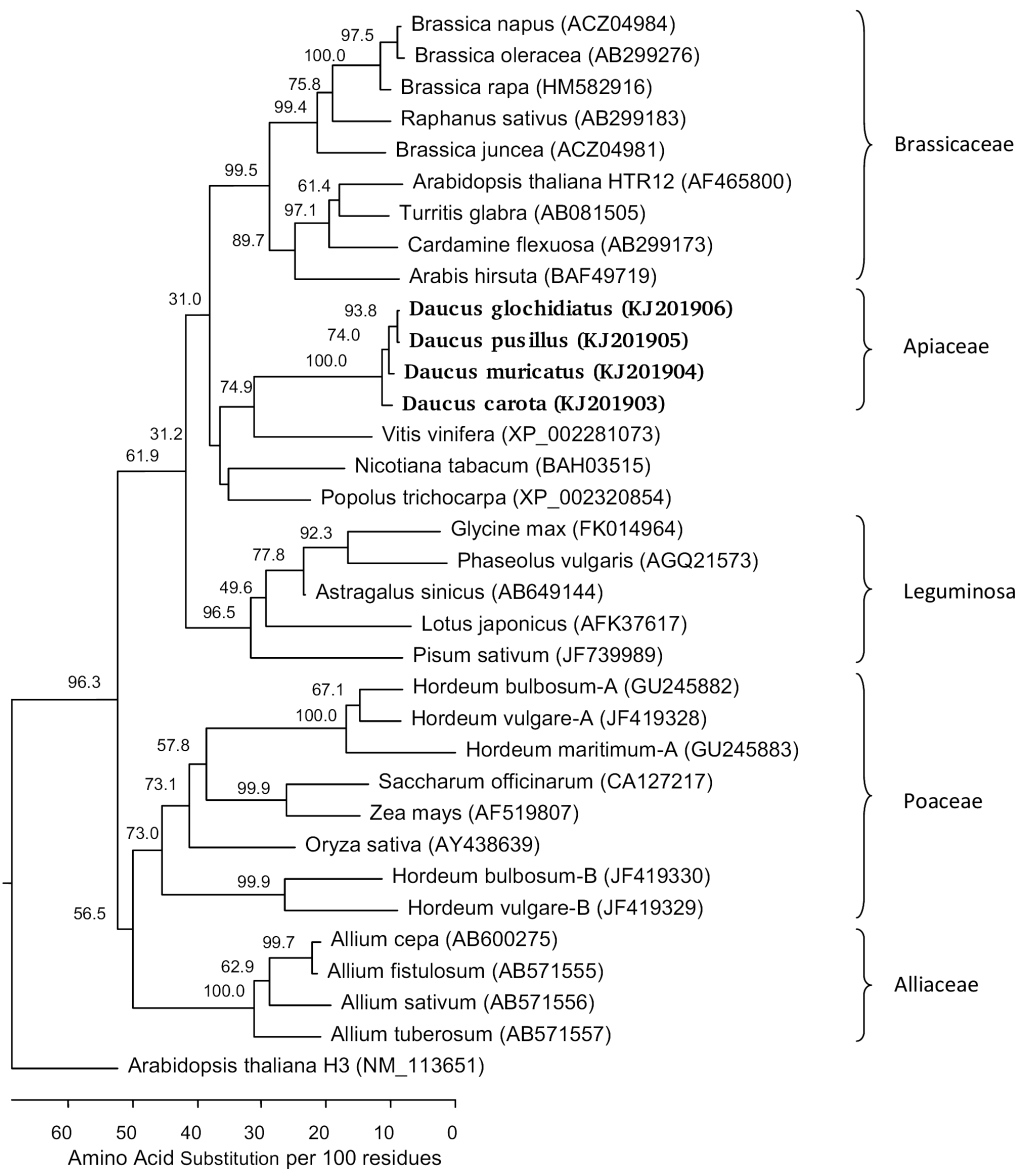
Phylogenetic tree of the deduced *Daucus* CENH3 proteins (printed in bold letters) and a selection of plant CENH3 proteins representing monocot (Alliaceae, Poaceae) and various dicot plant families including Leguminosae and Brassicaceae. Canonical histone H3 of *A. thaliana* was used as an outgroup. For each amino acid sequence, the NCBI accession number is indicated in parentheses. Multiple sequence alignment was performed by ClustalW using the Lasergene (DNASTAR) software package. A phylogenetic tree was constructed using the Kimura distance formula to calculate distance values and bootstrap analysis (10,000 replicates). Numbers indicate bootstrap replication, and branch length is scaled below the tree indicating the number of amino acid substitutions per 100 amino acids.

When the *Daucus* CENH3 proteins were aligned for phylogenetic analysis with those from various other monocot and dicot plant species, and *A. thaliana* canonical histone H3 as an outgroup, the carrot CENH3s formed a specific *Daucus* (Apiaceae) clade which was relatively closely located to CENH3s from grape, tobacco and poplar (Figure 2). The nucleotide identity values of the comparisons to *V. vinifera* CENH3 coding sequence were in a range of 66.4% and 67.4% (Figure S2), and the amino acid identity was about 67% to 69% depending on the *Daucus* species (not shown). Most characteristic for this comparison was the lack of ten consecutive amino acids in the hypervariable N-terminal tail domain of *Daucus* CENH3s, whereas the putative centromere targeting domain (CATD) was exactly of the same length (Figure 1). The CATD is composed of the loop 1 linker and α-2 helix of the histone fold domain of the C terminal part of CENH3 and is important for binding of CENH3 to centromeric DNA [37]. Its role has been documented also for higher plants like *A. thaliana* [38]. A low degree of amino acid identity of *Daucus* CENH3s to *A. thaliana* H3 was found, and also to the identical *D. carota* canonical H3 sequence identified by bioinformatic mining in the *Daucus* transcriptome (result not shown). Overall, these finding as well as the phylogenetic arrangement of the sequences among some other dicot species suggest, that the deduced *Daucus* CENH3s are authentic CENH3 homologs.

### Transcriptional Analysis of *Daucus* CENH3 Variants

**Figure 3:**
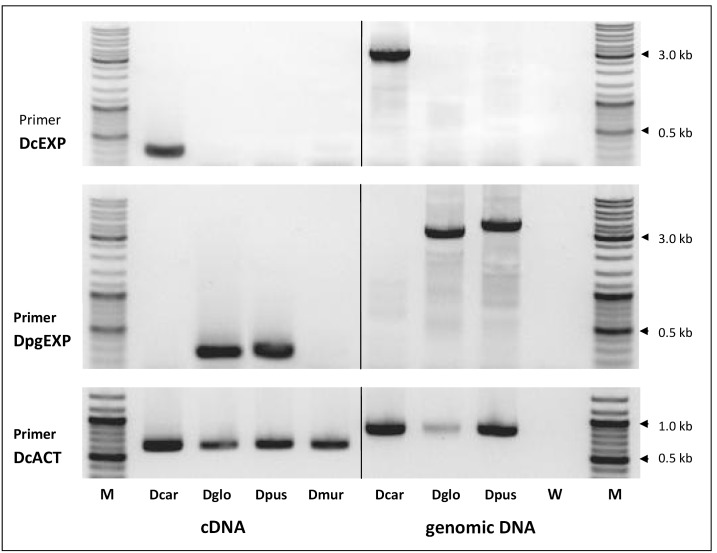
RT-PCR-based transcriptional analysis of CENH3 in *D. carota* (Dcar), *D. glochidiatus* (Dglo), *D. pusillus* (Dpus) and *D. muricatus* (Dmur) with gene-specific primer pairs designed for *D. carota* CENH3 (DcEXP) and CENH3s of *D. pusillus*/*D. glochidiatus* (DpgEXP). For details, see text, and for position of primers, see Figure S1. For the reference gene *β-actin* the primer pair DcACT was used. Positive control is genomic DNA of *D. carota, D. glochidiatus* and *D. pusillus,* and negative control is water (W). Size standard (M) is the Gene Ruler DNA ladder Mix (Thermo Fisher Scientific).

The sizes of the PCR products obtained after cDNA-PCR with the single universal primer pair DCEN2 appeared to be similar among the different genotypes, and also the clones obtained from individual accessions of each species did not indicate so far the possibility that alternative splicing might have been occurred. However, we wanted to include in this study some transcriptional analyses of different *Daucus* CENH3 sequences, to examine the possible existence of additional transcribed alleles, which have not been revealed by the cloning procedure used. Therefore, a set of species-specific internal PCR primers was developed for RT-PCR analysis of each CENH3 variant. Using sequence differences present at nucleotide positions 86 and 88, forward SNP primers were designed for *D. carota*, *D. glochidiatus* and *D. pusillus* (Figure S1). In *D. muricatus* it was not yet possible to develop a SNP-specific primer pair. Due to the high similarity of *DgCENH3* and *DpCENH3* the same primer pair (DpgEXP) was chosen. As shown in Figure 3, the *D. carota* - specific primers (DcEXP) produced a single fragment of the expected size of 315 bp only in *D. carota*, but not in any of the other species. Vice versa, the RT-PCR with the DpgEXP primers displayed species-specific transcription in both *D. glochidiatus* and *D. pusillus,* but not in *D. carota* and *D. muricatus*. We can therefore exclude, that tetraploid *D. glochidiatus* cells contain the same expressed CENH3 variant of *D. carota.* Although we assume the existence of a single transcript of the CENH3 gene in this polyploid species, interpretations regarding the number of putative alleles and transcripts should still be done cautiously. Only a relatively small number of five individual clones have been randomly selected and sequenced after PCR-based cloning, and the presence of additional CENH3 alleles is possible. Hirsch et al. [22] identified two distinct CENH3 transcripts in allotetraploid *Oryza* species and were able to trace their origin back to diploid rice species known as putative progenitors. In *Brassica*, where the situation is more complex, up to four distinct CENH3 cDNAs were identified in individuals of each of the diploid species *B. rapa*, *B. oleracea*, and *B. nigra,* and the presence of multiple isoforms in allotetraploids derived from them suggest multiple CENH3 loci in *Brassica* [27]. In natural allopolyploids of wild rice and tobacco obviously all CENH3s from each genome retain their expression, whereas in soybean with its putative polyploid genome structure only a single transcribed homolog of CENH3 was found [28]. In *Daucus* it would be interesting to conduct interspecific crosses i.e. *D. carota* x *D. glochidiatus* followed by transcriptional analyses and immunostaining experiments on chromosomes of hybrid embryos.

### Chromosome 9 Encodes CENH3 of *D. carota*

**Figure 4:**
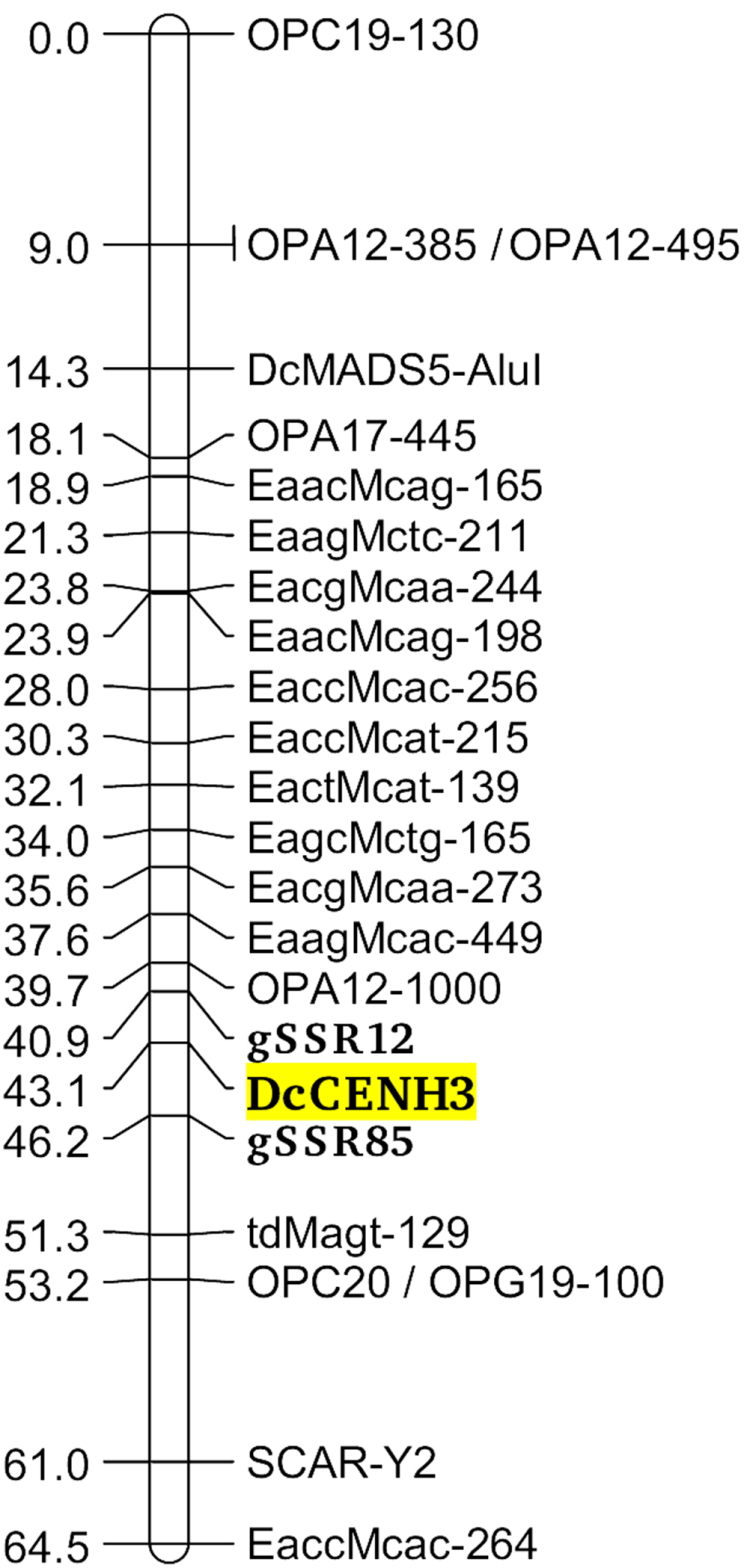
Genetic map of the carrot chromosome 9 (corresponding to linkage group 7) with the calculated position of the *DcCENH3* gene mapped through the DCEN-SSR marker. Scale: centiMorgan (cM).

For localization of the CENH3 gene in the carrot genome through linkage mapping, a previously constructed genetic map of the carrot progeny DM19 was used. This well-saturated map has been constructed on the basis of 285 molecular markers and has already been used for mapping of several genes involved in flowering characteristics [Budahn, unpublished]. Based on the compound dinucleotide SSR motif (CT)_14_ CCC (CT)_3_ TT (CT)_6_ present in the second intron of the genomic sequence, a specific PCR primer pair was developed (DCEN-SSR, Figure S3A). The CENH3-specific fragments segregated in DM19 progeny as a co-dominant marker (segregation type ‘hk×hk’ according the JoinMap format [35]), and the CENH3 locus was mapped on the carrot linkage group 7, which has been designated as chromosome 9 after the integration of genetic and physical maps of *D. carota* [13]. As shown in Figure 4, the location of the CENH3 gene was calculated between two anonymous genomic SSR markers (gSSR12, gSSR85) in the bottom part of the chromosome. According to marker information of the dense carrot linkage map presented by Cavagnaro et al. [6] there might be a tight genetic linkage of DCEN-SSR to a structural key gene involved in carotenoid biosynthesis (ζ-*carotene desaturase*, *ZDS2* [39]) located in the middle of the interval between the markers gSSR12 and gSSR85. This finding suggest that the repeat motif within *DcCENH3* might also be useful as a highly informative molecular marker for association studies targeted to carotenoid biosynthesis in carrots.

### Visualization of Centromeres with a Carrot CENH3-specific Antibody

**Figure 5:**
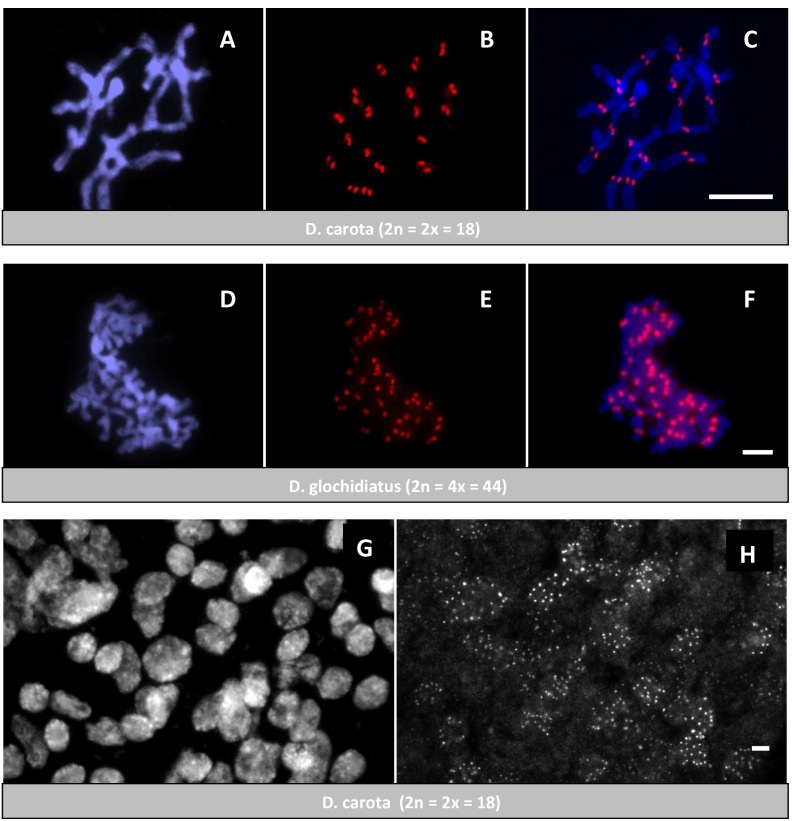
Immunostaining of *Daucus* root tip cells using anti-*DcCENH3* antibody. (**A**–**C**) *D. carota* (2n = 2x = 18) metaphase chromosomes, (**D**–**F**) *D. glochidiatus* (2n = 4x = 44) metaphase chromosomes, (**G**, **H**) interphase nuclei of *D. carota*. A, D and also G are DAPI-stained chromosomes, B, E and also H are CENH3 immunosignals, C and F are merged images. Scale bar 5 µm.

**Figure 6:**
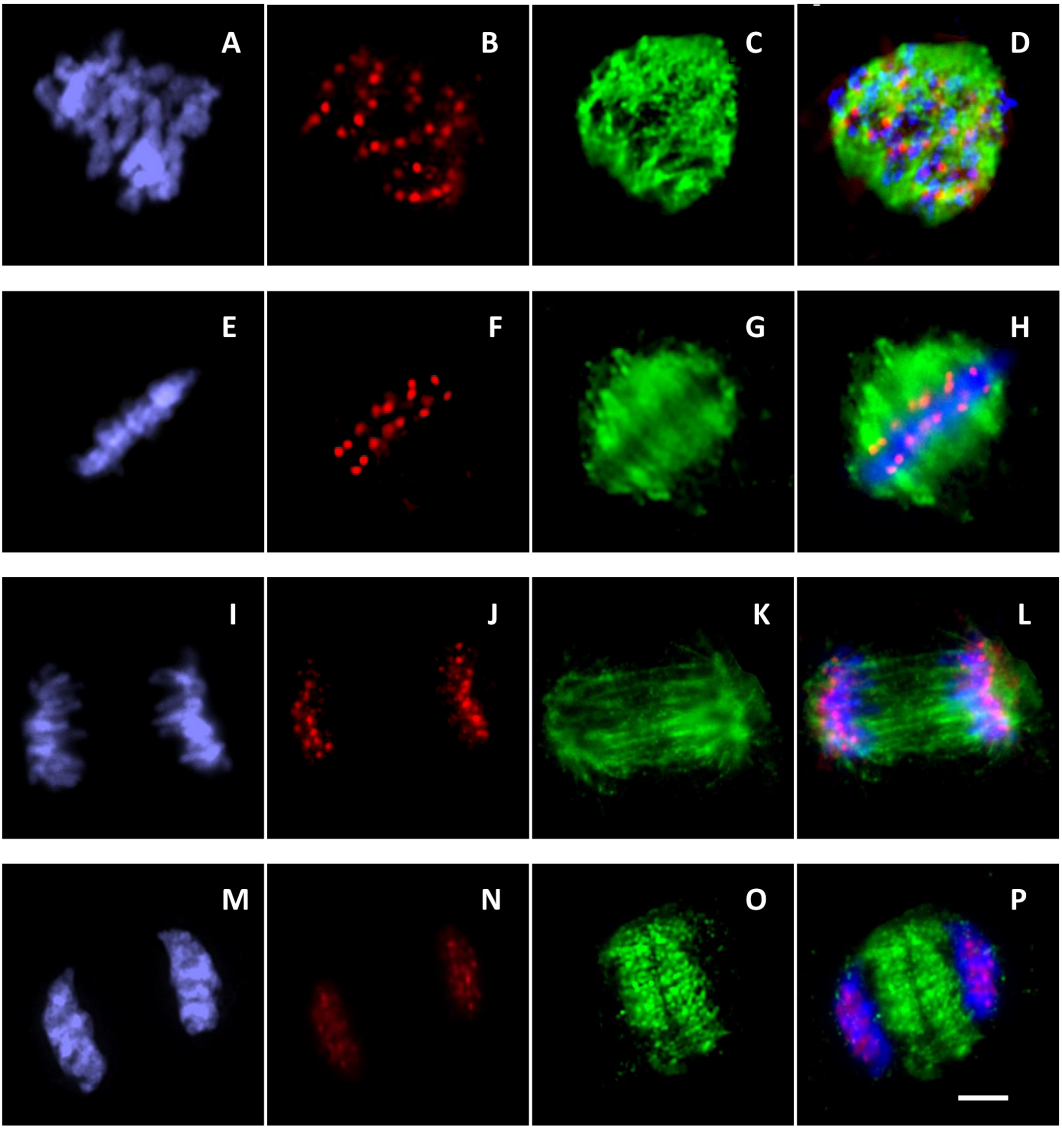
Double-immunostaining of root-tip cells of carrot (*D*. *carota*) at different stages of mitosis with antibodies against carrot CENH3 (in red) and α-tubulin (in green). Chromosomes are counterstained with DAPI (in blue). (A–D) prophase, (E–H) metaphase, (I–L) anaphase, (M–P) telophase. Scale bar 10 µm.

To verify that *DcCENH3* proteins localize to *Daucus* centromeres, immunofluorescence experiments were performed on mitotic chromosome preparations. Anti-*DcCENH3* antibody staining in root-tip cells of *D. carota* showed, that signals were exclusively located at the centromere regions of all 18 metaphase chromosomes (Figure 5A–C) providing direct *in situ* evidence for centromeric localization. As shown in Figure 5D–F, also tetraploid nuclei of *D. glochidiatus* appeared to be stained at the centromeric regions, indicating the cross-reactivity of the *D. carota* antibody with CENH3 of other *Daucus* species. Signals were also visible during interphase (Figures 5G and 5H) of carrot mitotic cells. After double immunostaining with anti-CENH3 and anti-α-tubulin, CENH3 signals were present in all stages of *D. carota* root cell mitosis (Figure 6). During anaphase, the antibody signals were located mainly at the tip of microtubule bundles attaching on the leading portions of the chromosomes in opposite orientations (Figure 6I–L). At telophase, the α-tubulin signals were mainly located in the equatorial plane, although the CENH3 signals remained at the two cell poles (Figure 6M–P). The cross-reactivity of the carrot CENH3 antibody with centromeres of distantly related *Daucus* species was not unexpected considering the very small sequence difference of a single amino acid in the N-terminal sequence of the deduced CENH3 protein used to create an antiserum. In several cases, anti-CENH3 antibodies were used to recognize CENH3s of more or less closely related species. The wide cross reactivity of antibodies raised against rice CENH3 [21] has been demonstrated in other *Oryza* species [40] and several other *Poaceae* species such as barley [41], wheat [42], and rye [43]. Wide cross-reactivity was also observed between different *Brassica* species [26] and several *Allium* species [25]. From our results with *Daucus* species it can be assumed that the *DcCENH3* antibody might be eventually also useful for the characterization of CENH3s of members of other genera of the Apiaceae plant family such as fennel (*Foeniculum*), celery (*Apium*), or parsley (*Petroselinum*). Work is in progress to confirm this assumption, and to clone the involved genes for further functional studies.

## Supporting Information

Figure S1 **Nucleotide sequence alignment (ClustalW, Lasergene) of the CENH3 coding sequences of** ***D. carota*** **(Dc),** ***D. glochidiatus*** **(Dg),** ***D. pusillus*** **(Dp), and** ***D. muricatus*** **(Dm).** Sequences of PCR forward primers used for species-specific RT-PCR are labeled by red- (*D. carota*) or blue-edged boxes (*D. pusillus*, *D. glochidiatus*), and (identical) reverse primer sequences are marked by a green-edged box.
(TIF)

Figure S2 **Phylogenetic tree built on the basis of cDNA nucleotide sequences of CENH3 variants identified in the four** ***Daucus*** **species of this study and two published CENH3 sequences (** ***N. tabacum,*** **NCBI acc. No. BAH03515;** ***V. vinifera*** **, XP_002281073) showing the highest similarity to** ***Daucus*** **CENH3s after multiple alignment of various plant CENH3 proteins (see** **Figure 2** **).** Sequences were compared by ClustalW (Lasergene). Branch length is scaled as number of substitutions per 100 nucleotides. In the table below the nucleotide sequence identity is shown (%) for the six sequences of the dendrogram shown above.
(TIF)

Figure S3 **(A)** Result of the alignment of the *D. carota* CENH3 coding region (cDNA sequence) with the genomic DNA (gDNA sequence) showing the intron-exon-structure of the *DcCENH3* gene. The position of a PCR primer pair designed for genetic mapping of *DcCENH3* (DCEN-SSR-F/-R) is also shown. **(B)** *DcCENH3* cDNA sequence and the deduced amino acid sequence. The positions of introns are marked by a red arrow.
(PDF)
