# Supplementary figures and images for: Characterization of Centromeric Histone H3 (CENH3) Variants in Cultivated and Wild Carrots (Daucus sp.)
Source: PLoS One. 2014 Jun 2;9(6):e98504. doi: 10.1371/journal.pone.0098504 (PMC4041860; doi:10.1371/journal.pone.0098504)

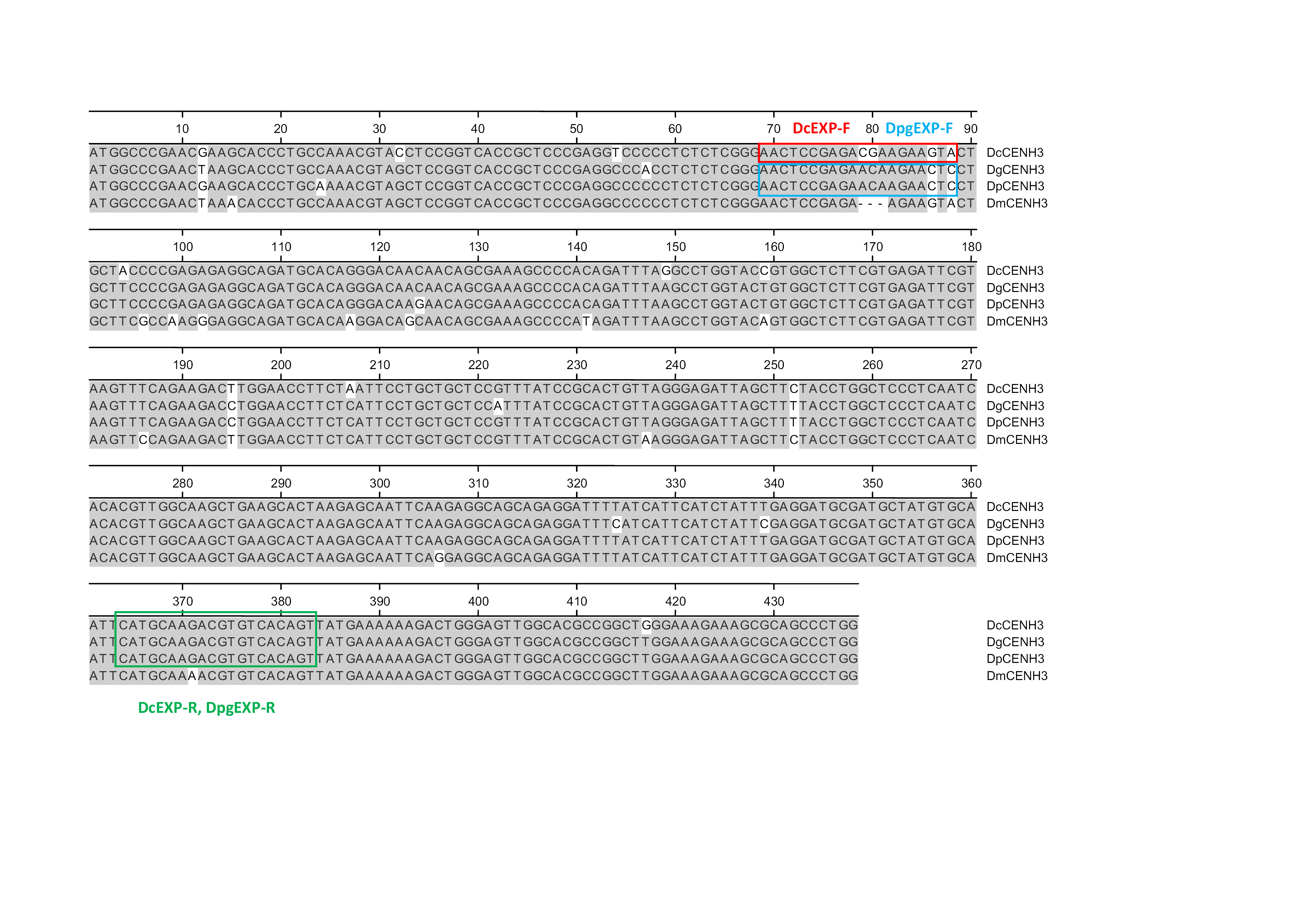

Supplement: Figure S1 — Nucleotide sequence alignment (ClustalW, Lasergene) of the CENH3 coding sequences of D. carota (Dc), D. glochidiatus (Dg), D. pusillus (Dp), and D. muricatus (Dm). Sequences of PCR forward primers used for species-specific RT-PCR are labeled by red- (D. carota) or blue-edged boxes (D. pusillus, D. glochidiatus), and (identical) reverse primer sequences are marked by a green-edged box. (TIF) [file pone.0098504.s001.tif]

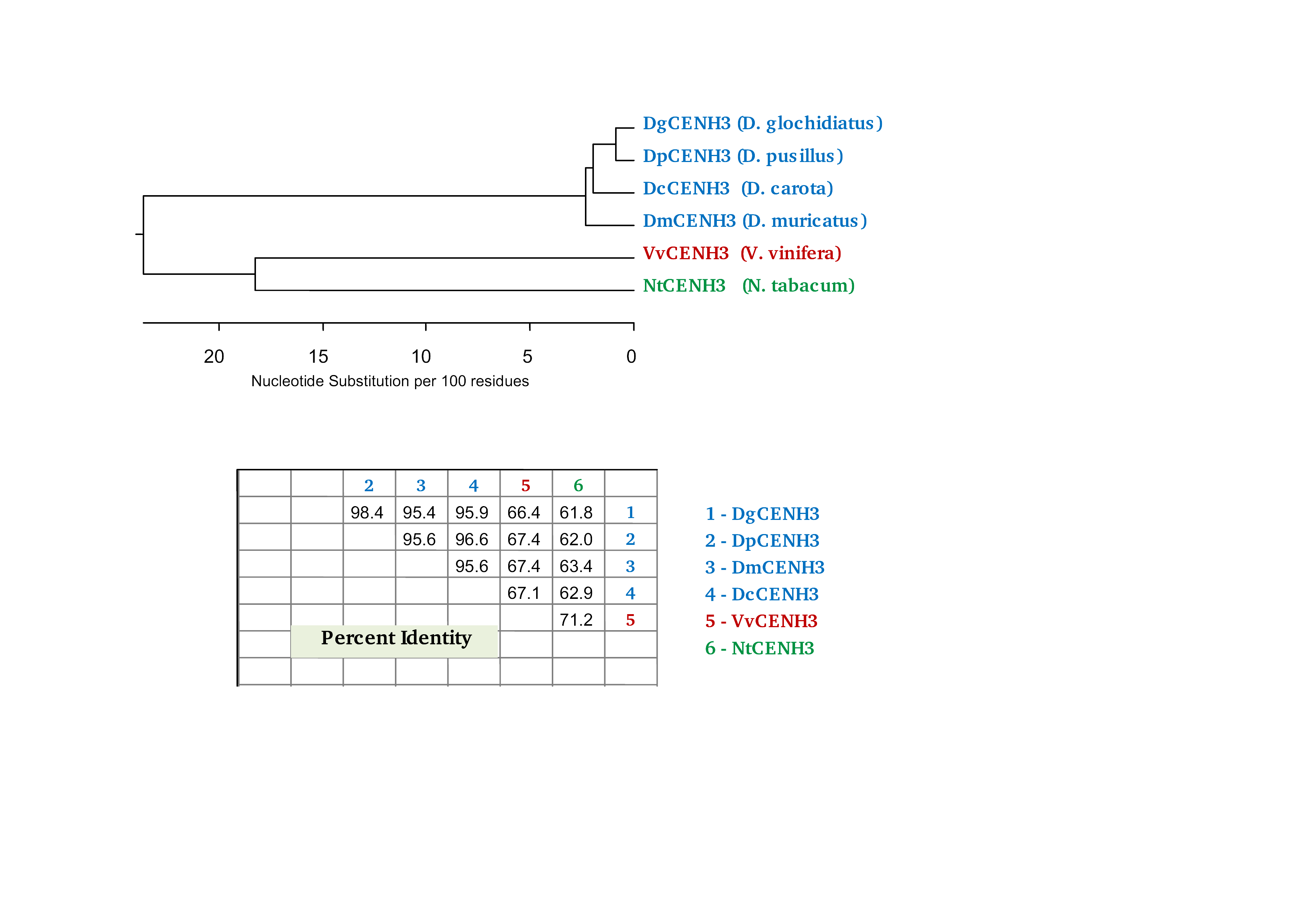

Supplement: Figure S2 — Phylogenetic tree built on the basis of cDNA nucleotide sequences of CENH3 variants identified in the four Daucus species of this study and two published CENH3 sequences ( N. tabacum, NCBI acc. No. BAH03515; V. vinifera , XP_002281073) showing the highest similarity to Daucus CENH3s after multiple alignment of various plant CENH3 proteins (see Figure 2 ). Sequences were compared by ClustalW (Lasergene). Branch length is scaled as number of substitutions per 100 nucleotides. In the table below the nucleotide sequence identity is shown (%) for the six sequences of the dendrogram shown above. (TIF) [file pone.0098504.s002.tif]
