# Supplementary material for: Characterization of Centromeric Histone H3 (CENH3) Variants in Cultivated and Wild Carrots (Daucus sp.)
Source: PLoS One. 2014 Jun 2;9(6):e98504. doi: 10.1371/journal.pone.0098504 (PMC4041860; doi:10.1371/journal.pone.0098504)
Supplement: Figure S3 — (A) Result of the alignment of the D. carota CENH3 coding region (cDNA sequence) with the genomic DNA (gDNA sequence) showing the intron-exon-structure of the DcCENH3 gene. The position of a PCR primer pair designed for genetic mapping of DcCENH3 (DCEN-SSR-F/-R) is also shown. (B) DcCENH3 cDNA sequence and the deduced amino acid sequence. The positions of introns are marked by a red arrow. (PDF) [file pone.0098504.s003.pdf]

Exon 1 **DCEN-SSR-F**

5' ATGGCCCGAACGAAGCACCCTGCCAAACGTACCTCCGGTCACCGCTCGCGAGGTCTCTCTCCCTCACACA 70

5' CTTATTTTATTAATTTAATTTGTACCGTTTTTCAATTGCTCTTTCCAAGCCCTAAATTATCTTCCTCCTT 140

Exon 2

5' TTATGTTACAGGTCCCCCTCTCTCGGTAT**CTCTCTCTCTCTCTCTCTCTCTCTCTCTCCCCTCTCTTTCT** 210

DCEN-SSR-R

5' **CTCTCTCTCT**GAAATTGGAGTTATATAAAATTTTGAATGCTGATGAGTATTTATACAGGGAACTCCGAGA 280

Exon 3

5' CGAAGAAGTACTGCTACCCCGAGTAAGTCTATAATCTTCGTATTGTTCGAGTTAGGGATTCAGTTTTTGT 350

5' TAGTCAAATGAAACTGATTTCGTTGATATAGGAGAGGCAGATGCACAGGGACAACAACAGCGAAAGCCCC 420

Exon 4

5' ACAGATTTAGGCCTGGTACCGTGGCTCTTCGTGAGATTCGTAAGTTTCAGAAGACTTGGAACCTTCTAAT 490

5' TCCTGCTGCTCCGTTTATCCGCACTGTAAGTTATCGCTACTTGTTTAATTTCTGTTTATTTTTGTATTTG 560

5' GTTTGGTATTCGGATATAATTGCCCTTAGTAATGGATGGAAATTTGAATTATATCGACTGTGGAAGTGTG 630

5' AATGGATAGTAATTAAAGTTTAAATGCTTTGAACCAGAATGGCACGGGATATTGTGGTATGACTTAATTG 700

5' AAGTTGTACCTAATAGGTTCTTTTTTGTTGCATACACTCAAGATCTGCTAGCTTCTTTATATAGTTAGAT 770

5' GCACTGATTTATTTTCATGACTAACAATGTTAATGTAGGTATTTTAAATGTTCTCAAGAAAACTTTTTAT 840

5' TTGGTGGCTAATATGGAAAAGTTCTTTTCTTATTGAACTATTATTTGTTGAAAATTTACCCCCCAAATCT 910

5' GGTTTTCTTTTCGTGTATGATAGAAGTTATTTGGCAGAATGTGTTATTATGTGAATATCAAACAACCTGA 980

5' GCAACAGAATACTGATTTAATATGGACTATAATTGTAAATCTATTTGAAATTGTCTGTAGTATCATGAGT 1050

5' GACTAGAATATTTTGTGCATATATGTACGGCACCTTTGTAACCTACAACTCTCATTTTGAAATGGTAATA 1120

5' GTAATATCAAATATGTCTACTTGTGAATTCAGTTGGTATTTGATGTCATAATAATTGTTAACAGGTTAGG 1190

Exon 5

5' GAGATTAGCTTCTACCTGGCTCCCTCAATCACACGTTGGCAAGCTGAAGCACTAAGAGCAATTCAAGAGG 1260

5' TATTTAAAAATTCTGTCCAACTAAATTCTGTTGTCCAGTACTACTTTGTCATTTTTTTTAAAGCATTATA 1330

5' ACTGAATTAGAAACACTATCGAATGACCAAGACTTTAACAGTACTACTTTGGGTACCCCTTTCCGGCTTT 1400

5' CCTATATATAATATAAAGTTTGGCTCTGGAAGAAACACACTGCATATAGGCAAAGGCTTAATCAGCTATT 1470

5' TAATGTTATACTTGTCACATTTAATGCCATAAGCATACATATCAAATTTAAAAGACTTGACCAGCTTATT 1540

5' TTGTTTCCAAAATCATACAGAAGAAATACCATTATTTTACTCACTAGATATTGTAAATTTTCTTATGAAG 1610

5' CTTCCTTTGACTTATTGAAAAGATATACGATAATCCACTTAAGATAAATCATAATCCTTAGAATTGTAAA 1680

5' AACAAATGATTGTGCATTTGTGCCCCTTCCTGCAATAGACGACATATTAGCTTCATGTAGAAGCCCCTTA 1750

5' CAAATATCAAGTATTAGTACAGAATTATGCATAATGACTAACCCTTGACAATTGGAAGTTTGCAATTATT 1820

5' TCATTGTGGAATGAGTTTTTTGTTTCAAAAATTTATCTCTTGTGGAACAAAGTAAGTAAAATTTTATTTT 1890

5' ATTATGAAACCAAATAACTATGTTAGTTATTTTATTTTAGAGGTTGTACATTTGACATAGATGAGCATGA 1960

5' GATAACAGAGCAAATAATAATTTCAAATGATATACCGCTCTTTTTTTTATACATAACTTCTTCCATTATC 2030

5' GCATTCTAGGAACTGCATCTCTAACCGCATATTTTTTTGTTTAGATCAGTAAAATCGATAACCCCCGATT 2100

5' TTTTCGTAAACTATTTCCTTTCTCTACATGCTAGTATATAGAGTTTTCCCTTTCTTAGCTAGGCTGTTTG 2170

5' CATCTTTGTACTTTTAAATCTGCTCTCGAAATGTTTTTTGGAGATATTCTCTGATGTATAGAGACTTAAT 2240

5' ATCCTGGCTCACAGTTATAAATTTATTAGTCATTTTTTTGGTTATAATATATTTACAGTACCTGTGTTTG 2310

5' AAGTTGTTTAAATATATTTCCAGCAATGAATATCTTTTCTTTATCTGTTTGTTTATGGTTTTATCGTTGG 2380

5' TCGTGTTTTTAGTTTTGGTCCTTCAGAGGGTAAATTGTCATGATTTTCAATAATTATAATTGTATATATT 2450

5' GTACTTCTCTAGATAAGAGTTGACAAAGATAACATGAAACAAAAAAAGGTTCTTTCAAAGCATTGATATA 2520

5' ATCTCTGTTCTTTCAAAGTGCCATCATCATGTTTTTTAGATATGATCTCTTTTACTTATGAAGGCCCAAT 2590

5' GTCAAATAGTTACACAGTGGCTCTGAAGCAATCTGAATTCCAGGACTCAATTTACCAATATACAAAATCT 2660

5' GTGCTCTACAAATCTGTTTTTGTGCTTGCAGAGTTGCAGTAATGTTCTGAGTGGAGTATCTTAGTCCTGC 2730

5' AAACCTTTCCACTAAACTCACGCCAACACAAGTCACTACTTACATCGTTGCATATTTAAATGTCTTATTA 2800

5' AGTTCTTTTAGTTACTTCTTAGGTCTTTTACAAGTTAAATTCCAAAAAACACCTCCCAGACATGATAAAA 2870

5' GTGGTATTTCTCTTTATATCTTTAAGATTTATTACAAAAAGTGGTGTTTCTCTTGAATCCACGATTGTTC 2940

5' TTCCTTATTTTCTGCTGAAATTCTCACGGCTACTTGTTTGTGTTTAAATTCAATGATATAAATTGGAATT 3010

5' GCATATCTGAATTGCCCTAATTGTTTAGGTTCATATTGAATGATAAATTGATAACAAGTGGTGTTTTTCT 3080

5' TTATACCTATATAAAAATTTCTTTCAAATCTATGATTTATACTTCCTTATTCTCTGCTGAGATTCTTGCG 3150

5' GCTACTTGTTTGGGTTTAGATTTTATGATATTATTTGGGCGTGTATTTCTGAATTTCCTTTGTTGTTTGG 3220

5' GTTCATTTTACTTGAATTCTATGTGTTTAATTGAGCAGTTTATGTTGAGTGTGGTATTTTCAAGAACTGG 3290

5' CTCAAGAAAACAAGAAAACCGGAATCAGAACGGATCAACCAACTTCAATTAAAGACGCAGGAAAAATACA 3360

5' TAATTCAAACGATTAGGACAAGTAAAAGATAATTCAAATAATTTCATTCGATCATATATGCATTTTAGCT 3430

5' GGCCAGCAGCAATTCTATACTGTGAGAATTCAGATTCTAAATTGTATGTTTTTTTAGTAAAGGTACTGTT 3500

5' CATTTTGAAGTAGTATTATATTTATAATATTCTTTGAGTTCTCTTGAAATATTCTGGGAATCTAATATAC 3570

5' TTGCAAAAGATATACTTTTACTTAGATGTAGCCATCTTTGCAGGCAGCAGAGGATTTTATCATTCATCTA 3640

Exon 6

5' TTTGAGGATGCGATGCTATGTGCAATTCATGCAAGACGTGTCACAGTTAGTAAGTGTAAATTGATGTTTT 3710

5' GACATTTTTGTTCTTATCAGCCATTGCATCCCCTTTGTCTCTTTATGTTGCATCCTGAAGTCATATTTAG 3780

5' AATATGGACTAGAGTTCGTGTTCTCTTAAATTTGCACATATATATATATTGAAGATCACAGATGACAGAA 3850

5' CCACTAGAGCATTGTACATCCTATTTCTTCTCTGTGTAGCATTTCTAAGAATTAGATTGACTTCATTAAA 3920

5' ATTGTAAGTAATCCCAGTTAGCCATTATAGTCTCCCTGAATGGCTGCTGCACCTGTGAATTCTTGCATTA 3990

5' CAGCTACTCACTCAACCTAGAGTTTAAATGACAAACACAATTTATAACCAGATCATGGTGACTGGCACTG 4060

5' GCCCAAAGCTTTGTTTAGTTGAATGAGGTATCATATACATGTAGAGATCAGCCACTACACGGGGGTTTTC 4130

5' TTTACCGTTTATTGTTTTTATCTGCTTTTTCATTGAGTTTGTAGAGTGTTGTAAAAAGCGGAAATCGGTG 4200

5' GAGCCACCGTCTGGCGATTAATAGATAATCGGGGATATTACGCTAGTTTTAAAGAAAAATGTTGTGATTA 4270

5' ATCGGTTTTTAAAAAATGAATCGGCAGAGTTCCTTGTAGCAATTAACCGGCCAAATCGGCAACTTTTAGA 4340

5' ACTGTGTTTTGTACTAGATGCAAATTGACTCAAGTAAGATTTTTTTAAGTTTTTACTAGGGTCACTATTA 4410

5' CTCTCTGAGCAAGCAGCTACTTATTCGTTGTTATTATTGTTATGGCTCACAGTGAAAAAAGACTGGGAGT 4480

Exon 7

5' TGGCACGCCGGTTGGGAAAGAAAGCGCAGCCCTGG

Figure S3 A

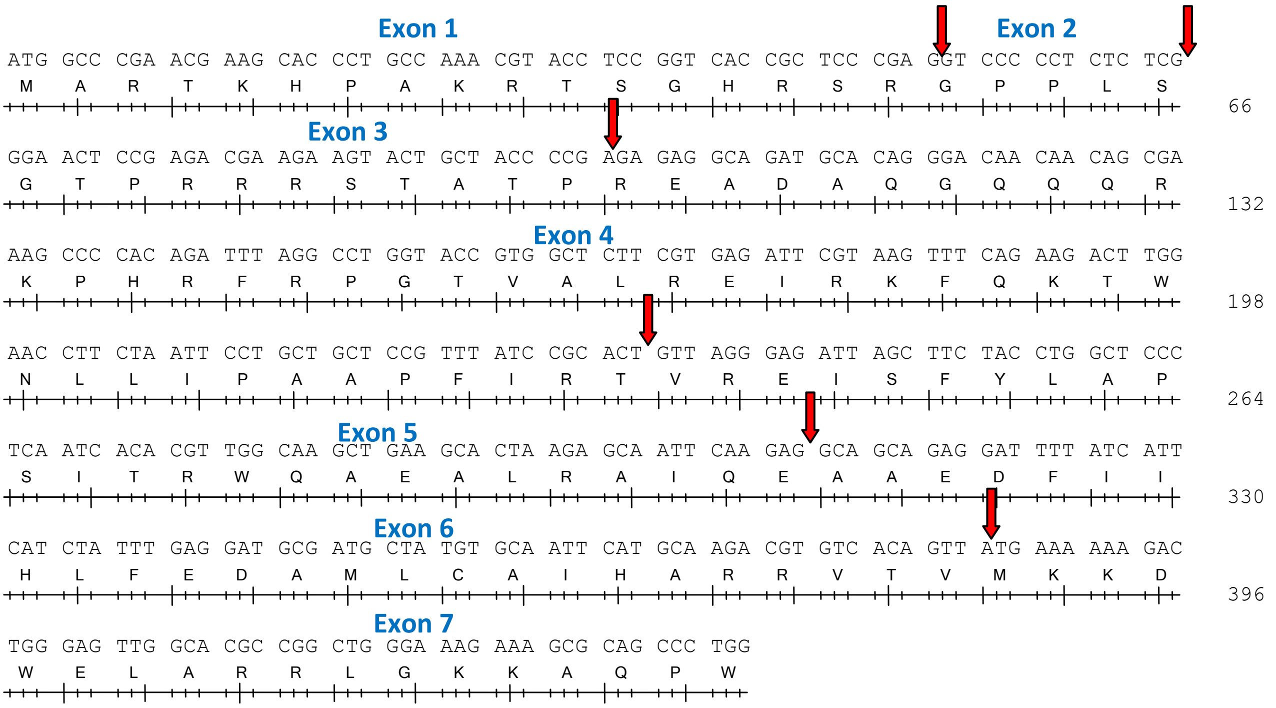
Exon 1
Exon 2
ATG GCC CGA ACG AAG CAC CCT GCC AAA CGT ACC TCC GGT CAC CGC TCC CGA GGT CCC CCT CTC TCG
M A R T K H P A K R T S G H R S R G P P L S
66
Exon 3
GGA ACT CCG AGA CGA AGA AGT ACT GCT ACC CCG AGA GAG GCA GAT GCA CAG GGA CAA CAA CAG CGA
G T P R R R S T A T P R E A D A Q G Q Q Q R
132
Exon 4
AAG CCC CAC AGA TTT AGG CCT GGT ACC GTG GCT CTT CGT GAG ATT CGT AAG TTT CAG AAG ACT TGG
K P H R F R P G T V A L R E I R K F Q K T W
198
AAC CTT CTA ATT CCT GCT GCT CCG TTT ATC CGC ACT GTT AGG GAG ATT AGC TTC TAC CTG GCT CCC
N L L I P A A P F I R T V R E I S F Y L A P
264
Exon 5
TCA ATC ACA CGT TGG CAA GCT GAA GCA CTA AGA GCA ATT CAA GAG GCA GCA GAG GAT TTT ATC ATT
S I T R W Q A E A L R A I Q E A A E D F I I
330
Exon 6
CAT CTA TTT GAG GAT GCG ATG CTA TGT GCA ATT CAT GCA AGA CGT GTC ACA GTT ATG AAA AAA GAC
H L F E D A M L C A I H A R R V T V M K K D
396
Exon 7
TGG GAG TTG GCA CGC CGG CTG GGA AAG AAA GCG CAG CCC TGG
W E L A R R L G K K A Q P W

Figure S3 B
